# Metabolic Connectome and Its Role in the Prediction, Diagnosis, and Treatment of Complex Diseases

**DOI:** 10.3390/metabo14020093

**Published:** 2024-01-26

**Authors:** Weiyu Meng, Hongxin Pan, Yuyang Sha, Xiaobing Zhai, Abao Xing, Sai Sachin Lingampelly, Srinivasa R. Sripathi, Yuefei Wang, Kefeng Li

**Affiliations:** 1 Center for Artificial Intelligence Driven Drug Discovery, Faculty of Applied Sciences, Macao Polytechnic University, Macau SAR 999078, China; p2214333@mpu.edu.mo (W.M.); p2314462@mpu.edu.mo (H.P.); p2215539@mpu.edu.mo (Y.S.); p2214950@mpu.edu.mo (X.Z.); p2314405@mpu.edu.mo (A.X.); 2 School of Medicine, University of California, San Diego, CA 92103, USA; sachinlingampally@gmail.com; 3 Henderson Ocular Stem Cell Laboratory, Retina Foundation of the Southwest, Dallas, TX 75231, USA; ssripathi@rfsw.org; 4 National Key Laboratory of Chinese Medicine Modernization, State Key Laboratory of Component-Based Chinese Medicine, Tianjin University of Traditional Chinese Medicine, Tianjin 301617, China; 5 Haihe Laboratory of Modern Chinese Medicine, Tianjin 301617, China

**Keywords:** metabolic connectome, network models, disease diagnosis, drug discovery, systems biology

## Abstract

The interconnectivity of advanced biological systems is essential for their proper functioning. In modern connectomics, biological entities such as proteins, genes, RNA, DNA, and metabolites are often represented as nodes, while the physical, biochemical, or functional interactions between them are represented as edges. Among these entities, metabolites are particularly significant as they exhibit a closer relationship to an organism’s phenotype compared to genes or proteins. Moreover, the metabolome has the ability to amplify small proteomic and transcriptomic changes, even those from minor genomic changes. Metabolic networks, which consist of complex systems comprising hundreds of metabolites and their interactions, play a critical role in biological research by mediating energy conversion and chemical reactions within cells. This review provides an introduction to common metabolic network models and their construction methods. It also explores the diverse applications of metabolic networks in elucidating disease mechanisms, predicting and diagnosing diseases, and facilitating drug development. Additionally, it discusses potential future directions for research in metabolic networks. Ultimately, this review serves as a valuable reference for researchers interested in metabolic network modeling, analysis, and their applications.

## 1. Introduction

Biological networks are widely used as graphical representations to describe and analyze biological systems. In these networks, graphs are used to represent biological entities, such as proteins, genes, RNA, DNA, and metabolites, as nodes. The edges of the network correspond to the physical, biochemical, or functional interactions between these entities [1]. Through analysis of these biological networks, the interrelationships between different biological entities can be revealed, including protein–protein, protein–DNA, protein–metabolite, and other associations. This allows the networks to capture the basic characteristics of biological systems and reveal the information patterns within them [2].

**Figure 1:**
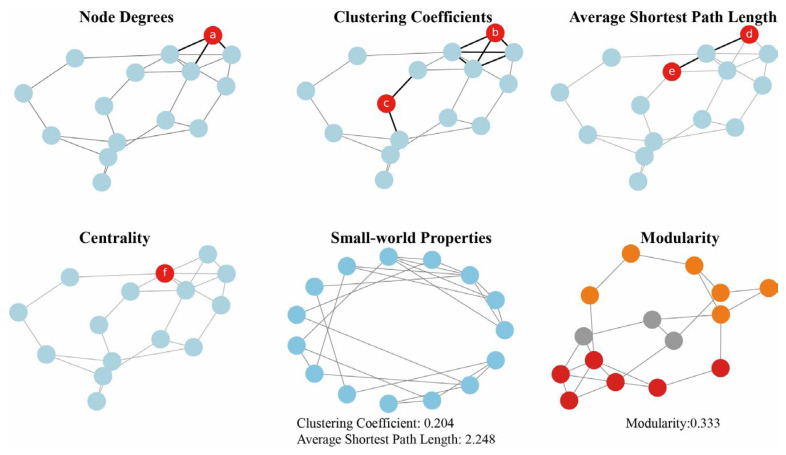
Network properties. In this example, node ‘a’ has a degree of 3. Node ‘b’ has a clustering coefficient of 1, and node ‘c’ has a clustering coefficient of 0. The average shortest path length between nodes ‘d’ and ‘e’ is two steps, passing through one intermediate node. Node ‘f’ contributes significantly to the centrality because it has a relatively large number of edges connecting it to other nodes. The small-world properties are measured by calculating the clustering coefficient and the average shortest path length. Each module in the modularity is represented by a different color.

In order to deeply understand and quantify the characteristics and behaviors of biological networks, researchers utilize a series of evaluation indicators (Figure 1). Indicators such as node degree, clustering coefficient, average shortest path length, and centrality are widely used to measure the degree of node connection, community structure, global connectivity, and node importance in networks [3,4]. Small-world properties describe the global structure of networks [5]. Additionally, modularity identifies functional modules and subnetworks, providing comprehensive evaluation for deeper understanding of biological system structure and function [6,7,8].

Currently, biological networks are classified based on different features and purposes. For example, protein–protein interaction networks describe protein interactions [9], gene regulatory networks reveal complex gene expression regulation mechanisms [10], and metabolic networks graphically represent metabolic processes [11]. Brain networks describe neuron and synapse interactions [12], while social networks represent social relationships between individuals [13]. Among these, metabolic networks have high plasticity and complexity as the basis of life activities and information transmission within organisms. They are complex network structures composed of interactions among multiple biological entities [11,14]. Metabolic networks are crucial in biological research to understand the complexity of biological systems and reveal interactions and regulatory relationships among different entities.

The main purpose of this review is to introduce the construction methods and existing applications of metabolic networks, as well as their potential application prospects. This provides a reference for researchers interested in this field. The remainder of this article is summarized as follows: The “Construction Methods of Metabolic Networks” section will introduce several common metabolic network models and their construction methods in detail. The “Application of Metabolic Networks” section will explore the applications of metabolic networks in various fields, including revealing disease mechanisms, predicting and diagnosing diseases, and drug development based on metabolic data. The final “Future Work” section will discuss possible future development directions for metabolic networks. Overall, this review aims to provide readers with a comprehensive and in-depth understanding of metabolic networks, hoping to stimulate further research interest and promote development in this field.

## 2. Construction Methods of Metabolic Networks

Metabolic networks can be represented by various types of relationships, including statistical correlations, causal relationships, biochemical reactions, and chemical structural similarities [14,15]. Statistical correlations and causal relationships are used to describe the relationships between molecules [16,17], while biochemical reactions and chemical structural similarities describe the interactions between molecules [18,19]. By constructing networks using these different relationship types, algorithms from network theory can be applied to metabolic networks to gain a more comprehensive understanding of metabolic processes [2]. The codes for constructing metabolic networks are provided in Table 1.

### 2.1. Correlation-Based Metabolic Network

**Figure 2:**
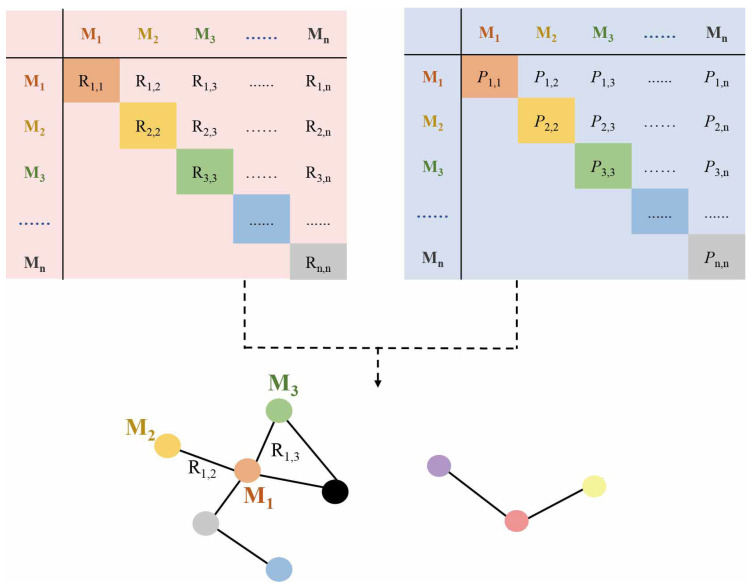
Creating metabolic network based on correlation analysis. Correlation-based metabolic networks use correlations among metabolites to establish connectivity relationships and simplify multidimensional data. R: correlation value; *P*: *p*-value; M: metabolite.

Correlation-based metabolic networks are widely used in metabolic research. These networks use the correlations among metabolites to establish connectivity relationships, simplifying multidimensional data while preserving most interpretive information (Figure 2) [22]. This method reveals coordinated behaviors between biological components and allows an analysis of network properties to better understand metabolite interactions and identify key metabolites in pathways [23,24]. Furthermore, correlation-based networks can also be applied to study metabolic disease pathogenesis and discover new treatments [22,25].

In a correlation network, the correlation value ranges from −1 to 1, with 1 representing a positive correlation, −1 representing a negative correlation, and 0 representing no linear correlation. The closer the correlation coefficient is to −1 or 1, the stronger the correlation, while values closer to 0 indicate a weak or no linear relationship. If the correlation value of two metabolites reaches a set threshold, a connection is established between them [26]. Methods to calculate metabolite correlations include Pearson correlation, Spearman rank correlation, distance correlation, and Gaussian graphical models [27,28,29]. Pearson correlation measures linear relationships, while Spearman rank and distance correlations assess monotonic relationships [27,29,30]. The Pearson correlation coefficients are obtained by calculating the covariance between variables divided by their standard deviations. The Spearman rank correlation coefficient sorts the values of the variables, then calculates the rank difference after sorting, and obtains it by dividing the covariance of the rank difference by the standard deviation. The distance correlation is obtained by calculating the distance covariance among variables divided by their respective standard deviations. Notably, a zero-distance correlation coefficient means variable independence, while zero Pearson and Spearman correlations do not necessarily mean independence [27,28].

However, due to the stringent metabolic control and extended reaction sequences present in metabolic networks, the use of Pearson correlation and Spearman rank correlation often results in highly interconnected and dense networks, complicating network analysis and interpretation [14]. Gaussian graphical models calculate partial instead of total correlations, correcting indirect effects to better reveal correlations in complex metabolism [31,32]. Importantly, observed correlations may be due to common influencing factors and do not necessarily represent direct causal relationships.

### 2.2. Causal-Based Metabolic Network

Causal relationship-based metabolic networks are complex biological networks that help us to understand the operating mechanisms of biological systems by revealing the interactions and effects between metabolites. Causal networks are graph models representing causal relationships, comprising variables and the causal relationships between them. The objective in constructing a causal network is to infer causal relationships between variables from observational data to better understand and predict system behavior [33,34]. The network consists of nodes, representing variables like genes, metabolites, and biological processes, and edges, representing causal relationships between variables that can be direct or indirect. A key feature of causal networks is discoverability, making them suitable for processing large-scale data with a limited understanding of interconnectivity [15].

Statistical methods using causal inference and discovery techniques are widely used in constructing causal networks to detect causal relationships between variables [35]. The causal inference model is a statistical framework used to infer causal relationships through observational data. This model applies statistical and causal inference principles, analyzing correlation, causal direction, and mechanisms to infer causal relationships [36]. Causal inference models include latent causal models and causal graphical models. Latent models infer relationships between latent and observed variables [37]. Causal graphical models use directed acyclic graphs to represent causal relationships between variables. They employ directed edges to represent causal relationships and determine conditional independence [38].

**Figure 3:**
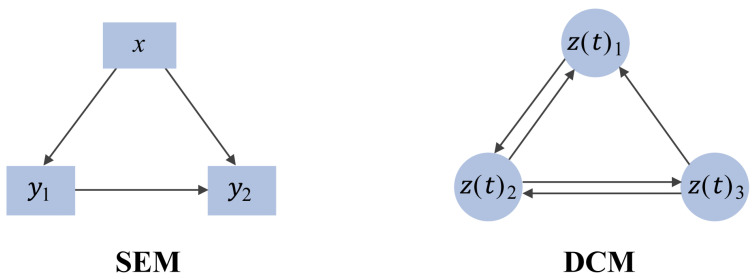
Structural equation model (SEM) and dynamic causal model (DCM). Among them, 𝑥 represents the independent variable, 𝑦 represents the dependent variables, and 𝑧(𝑡) represents the concentration of metabolites at time *t*.

In addition, structural equation modeling (SEM) and dynamic causal modeling (DCM) are also methods for causal inference (Figure 3) [35,39,40]. SEM is a multivariate statistical model that infers causal relationships among variables by modeling the relationship between observed variables and latent constructs, based on the covariance or correlation coefficient matrix [39,41]. Variables are manifest or latent. Manifest variables are directly measurable, while latent are indirect [42,43]. SEM can analyze direct and indirect effects among multiple variables, as well as relationships between variables and latent constructs [44,45].

In SEM, the relationships among multiple variables can be expressed by the following formula:

(1) y=λx+βy+ε

where 𝑦 represents the dependent variable, 𝑥 represents the independent variable, 𝜆 denotes the factor loading coefficient, *β* represents the structural coefficient, and 𝜀 represents the error term. Specifically, the factor loading coefficient 𝜆 describes the relationship between the observed and latent variables, and the structural coefficient *β* describes the relationship between latent variables. SEM can also interpret observed data, with model estimation results explaining variable relationships, the degree variables explain observed data, and the explanatory power of potential variables. This assists in understanding data generation mechanisms and causal relationships between variables [39].

DCM is a statistical model used to model time series data [40]. It considers temporal relationships and causal influences between variables based on dynamic system theory. This reveals the causal structure and dynamic characteristics of the system. By estimating model parameters, the connection strength and direction between variables are obtained, revealing the causal network between variables [46]. DCM can also be used for prediction and intervention analysis. Simulating and predicting the model provides understanding of the system’s dynamic evolution under different conditions [40]. The basic formula of DCM can be expressed as:

(2) $$z\left(t\right)=f\left(z,\theta \right)+\omega$$

where $z\left(t\right)$ represents the concentration of metabolites at time t, $f\left(z,\theta \right)$ represents the causal relationship between metabolites, θ denotes the parameter of the model, and ω represents the noise term. Among them, θ includes the causal relationship between metabolites and the time delay between them.

### 2.3. Pathway-Based Metabolic Network

**Figure 4:**
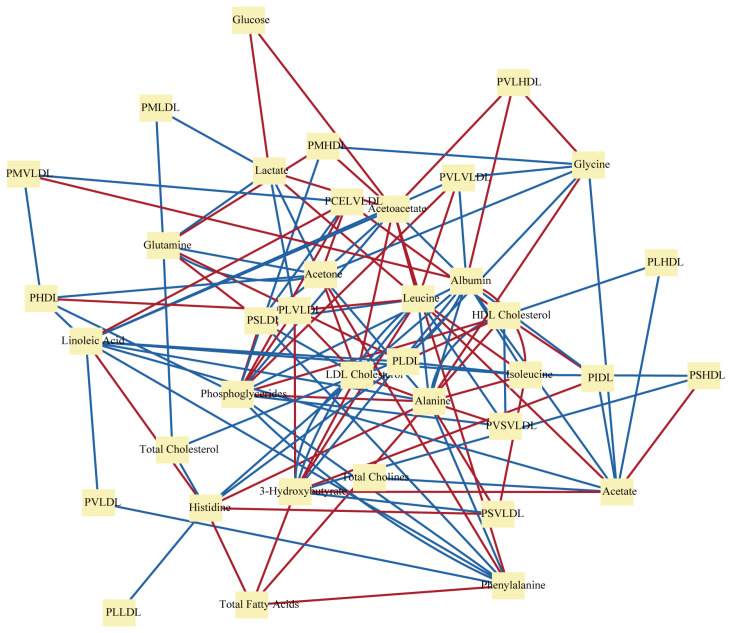
The metabolic network linking metabolic pathways and metabolites. Among them, blue represents negative correlation, and red represents positive correlation.

Pathway-based metabolic networks describe the interactions between biochemical reactions. These enzymatic reactions form the foundation of metabolic reactions within organisms, facilitating the synthesis, decomposition, and transformation of metabolites. Metabolites, including proteins, nucleic acids, sugars, lipids, and more, are chemical substances present within an organism. The complex metabolic network is formed by the biochemical reactions between these metabolites, interacting to maintain normal life functions (Figure 4) [47]. To better understand and utilize metabolic networks, it is necessary to select appropriate databases for data, prune networks for analysis, use algorithms to identify pathways, and develop computational methods to optimize pathways.

In designing metabolic pathways, database representation methods are used to describe the relationships between chemical reactions and metabolites. The two common methods are graph and stoichiometric matrix representations. Graph representations show topological connectivity using nodes for metabolites and edges for reactions. This visual representation intuitively displays topological and pathway structure, aiding understanding and analyzing pathway composition and function. Common graph-based databases include KEGG [48] and MetaCyc [49]. Stoichiometric matrices numerically describe quantitative stoichiometries between reactions and metabolites in rows and columns. This provides comprehensive quantitative information about their design, including reaction directionality, rates, and metabolite proportions. Common matrix-based databases include BiGG [50] and ModelSEED [51].

Network pruning is a commonly used technique to simplify complex metabolic networks during pathway design. This technique reduces complexity by removing irrelevant reactions or metabolites, thereby improving computational efficiency and design accuracy [52]. The goal is to remove components that do not significantly impact overall pathway performance, reducing computational and optimization complexity. This can be achieved by analyzing network structure and using dynamic simulation methods. Specifically, network pruning involves: A structural analysis of the network to identify important reactions and metabolites. Then, techniques like dynamic simulation and sensitivity analysis to evaluate each component’s impact on performance. Finally, the removal of components with less of an impact on performance to simplify the network. By deleting irrelevant components, network pruning reduces computational complexity and improves efficiency and accuracy of design.

To achieve metabolic pathway design, appropriate search algorithms and computational methods are also necessary. These algorithms aim to find optimal or near-optimal paths within large design spaces [53]. Search algorithms include breadth-first search [54], shortest path algorithms [52], etc., which are used to find the shortest or optimal paths in metabolic networks. Computational methods refer to the use of mathematical models and algorithms for calculations, such as metabolic network optimization based on flux balance analysis [55], a retrosynthetic search based on reverse synthesis [56], etc., which are used to calculate the efficiency of metabolic pathways and product yield. These search algorithms and computational methods can assist in the design and reconstruction of metabolic pathways to achieve specific metabolic product synthesis goals.

### 2.4. Metabolic Network Based on Chemical Structure Similarity

**Figure 5:**
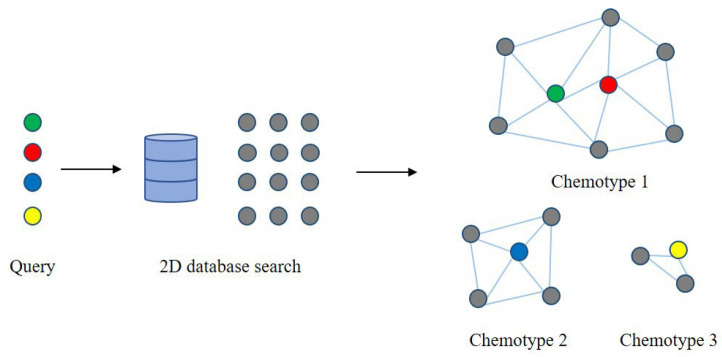
Chemical similarity networks. The reference compounds are identified from the bioactivity database using 2D similarity fingerprints of the query ligands. Then, the identified compounds are further clustered into chemical similarity subnetworks based on representative chemotypes. Among them, the colored nodes represent query ligands, and the gray nodes represent reference compounds.

Chemical structural similarity is a method of comparing and matching chemical molecules based on their structural characteristics. By comparing the structural features among compounds, the degree of similarity between them can be measured [57]. Metabolites with high similarity are often linked together, indicating that they may participate in similar metabolic reactions or pathways. The chemical and structural similarities among metabolites can then be converted into edges in the network to construct a metabolic network that reflects these similarity relationships (Figure 5) [58]. This network can reveal collections of metabolites with similar chemical structures, elucidating their functions and interactions in metabolic pathways.

Chemical structure descriptors play a key role in constructing metabolic networks. Chemical structure descriptors are numerical representation methods used to describe the structural characteristics of compounds [59]. Commonly used chemical structure descriptors include 2D and 3D chemical fingerprints [60]. Two-dimensional chemical fingerprints are feature vectors generated based on the 2D structural information of compounds, incorporating characteristics of compound connectivity, atomic type, and ring structure. These can be used to calculate the similarity among compounds and screen chemical libraries [61]. The Tanimoto index calculates shared features between 2D fingerprints to quantify similarity on a 0 to 1 scale, where values nearer 1 indicate higher similarity [61,62].

Three-dimensional chemical fingerprints are feature vectors generated based on the three-dimensional structural information of compounds, taking into account conformations, shape, charge distribution, and other 3D characteristics By calculating the 3D chemical fingerprint similarities among compounds, their structural similarity can be evaluated [63]. Euclidean distance evaluates the differences between 3D fingerprint vectors and is used to assess structural similarity, where less distance means more similarity [64]. 

Substructure matching is another common approach to determine similarity when constructing metabolic networks based on chemical structures [65]. This method can use SMARTS patterns or other expressions to describe and match substructures. SMARTS is a language that specifies atomic types, bond types, rings, and other structural features. By defining a specific SMARTS pattern, the substructure of interest and its position within a compound can be specified [66,67]. Compounds containing identical or analogous substructures are considered structurally similar, implicating comparable chemical properties and reaction behaviors. Incorporating compounds with shared substructures into metabolic networks allows inferences regarding analogous metabolic pathways or the functional interactions between them.

## 3. Application of Metabolic Network

Metabolites are more closely related to an organism’s phenotype than genes and proteins. Moreover, the metabolome serves to amplify potentially immeasurably small changes in the proteome and transcriptome, even those derived from minor changes in the genome. The health and disease states of the body can be more meaningfully characterized by the metabolic state of the human cells, tissues, organs, and the organism as a whole [68]. Abnormal metabolism either causes or results from complex diseases like hypertension, diabetes, cancer, and heart disease. Thus, adequately understanding human metabolism and metabolic interactions is a necessary step towards efficiently treating and diagnosing these complex diseases. However, metabolism involves countless individual reactions that are highly interconnected through shared metabolites [69]. Developing and applying metabolic networks plays a significant role in medical research, especially in elucidating disease pathogenesis, prediction, diagnosis, and drug discovery.

A metabolic network is a complex system of hundreds of metabolites and their interactions involved in energy conversion and chemical reactions within cells [70]. Exploring the function and structure of metabolic networks can provide insight into metabolic abnormalities and signaling transduction disorders in disease, and further revealing the strong link between disease and metabolism [71]. Systems biology and computational biology approaches are used to construct and model metabolic networks in analyzing them [72,73]. This elucidates pathway and interaction complexity, regulatory mechanisms between metabolites, and the rapid spread of single-node perturbations across the tightly regulated, simultaneous network [74,75]. However, such network complexity poses challenges for disease elucidation.

### 3.1. Metabolic Networks in Disease Mechanisms

Firstly, a strategy to compare metabolic networks in disease states and normal states followed by identifying changes in disease-related metabolic pathways is an essential way for discovering and confirming disease-specific metabolic abnormalities. These changes may include the depletion or accumulation of metabolites, alterations in enzyme activity, and the remodeling of metabolic pathways. Gaining a deeper understanding of these abnormalities can shed light on the pathogenesis of the disease. Agren et al. [76] used the INIT (Integrative Network Inference for Tissues) algorithm to infer active metabolic networks for 16 different cancer types by comparing them with 24 healthy cell types, thereby identifying the metabolic features of cancers. The serine, glycine, one-carbon (SGOC) metabolic network is implicated in cancer pathogenesis, but its general functions are unknown. Mehrmohamadi et al. [77] computationally reconstructed the SGOC metabolic network in cancer and normal tissues and then characterized its expression across thousands of cancer tissues. This study confirmed the heterogeneous and expansive functions of the SGOC metabolic network in human cancer. Currently, some metabolic network analysis servers can study the evolution and functions of metabolic networks using traditional and ‘omics datasets’ [78].

Metabolic networks represent cellular metabolism through lists of reactions occurring in cells [79]. These reactions have been associated with particular cellular compartments and further grouped into pathways. Certain metabolic pathways may play crucial roles in particular diseases or physiological states, and regulating metabolic pathways is essential for maintaining normal physiological states [80]. Metabolic networks integrate metabolomics and pathway databases. Network topology and metabolite flow analysis identify pathways and regulation implicated in pathogenesis, such as abnormal glycolytic pathways in tumor cells [81,82]. Moreover, understanding their regulatory mechanisms can be helpful in elucidating the dynamics of metabolic pathways and their abnormal regulation in disease, such as transcriptional regulation, translational regulation, post-transcriptional regulation, and feedback regulation of metabolites. 

Moreover, metabolites can be passed between compartments (e.g., mitochondria or cytoplasm) through transport reactions, thereby acting as signaling molecules involved in regulating pathological and physiological processes in cells [83]. The close interaction between metabolic networks and signal transduction networks can help reveal how metabolic abnormalities affect signal transduction and further understand the pathogenesis of diseases [84].

**Figure 6:**
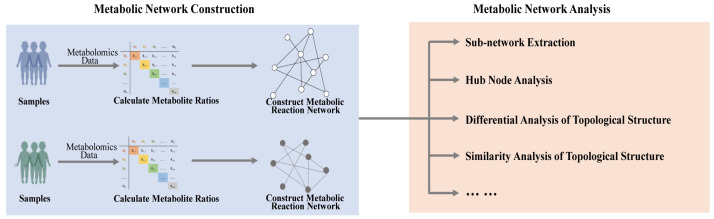
Metabolic network analysis provides a considerable tool for personalized medicine. Metabolic network could potentially be used to identify crucial metabolic network signals to reveal the mechanism of diseases, and further provide worthy information for personalized medicine.

Metabolic network analysis also provides a considerable tool for personalized medicine. By integrating clinical, genomic, and network data, one can predict drug responses and guide individualized treatment. This improves effectiveness and reduces side effects. Type 2 diabetes mellitus (T2DM) is recognized as one of the main threats to human health in the 21st century, emerging as a complex metabolic disease [85,86,87]. A study identified crucial network markers to predict personalized gliclazide responses [88]. The differential metabolic network construction (DMNC) method analyzed two T2DM metabolomics datasets. It defined a network biomarker to assess suitability for gliclazide therapy, showing a more stable performance than individual metabolites. Huang et al. [89] also provided a computational strategy for metabolic network construction based on the overlapping ratio for different dialysis patterns effect research in uremia patients. Above all, metabolic network analysis could potentially be used to identify crucial metabolic network signals to reveal the mechanism of diseases, and further provide worthy information for personalized medicine (Figure 6). Moreover, metabolic network analysis also enables us to research the intricate interactions between tumors and their microenvironment on multiple scales, and thereby generate patient-specific models [90].

The establishment and simulation of a metabolic network model can be beneficial to understand the pathogenesis of diseases. Multi-omics data integration has built dynamic models simulating pathway and metabolite changes in disease [91]. These models may predict disease progression, assess therapeutic efficacy, and further inform drug development. Moreover, metabolic networks do not operate in isolation, but are firmly intertwined with other regulatory mechanisms such as gene-regulatory or signaling networks. The integration of metabolic networks with other biological networks, such as protein interaction networks or gene regulatory networks, can provide a more comprehensive understanding of disease pathogenesis [92]. Drawing on biological information from diverse levels, multiple factors and complex interactions underlying disease can be further elucidated, improving comprehension of disease onset and progression [93].

The metabolism is involved in all aspects of human disease, and thus a reliable and complete human metabolic network is critical for elucidating correlations between human metabolism and disease. The generic human metabolic network Recon 1, comprising 1496 genes, 3748 reactions, and 1469 metabolites, has been the most-used human metabolic model in recent years. It has been applied to map transcriptomic data related to insulin sensitivity [94,95,96]. In this study, an analysis of expression profiles was performed during a dietary intervention program on the macrophages and adipocytes in tracked obese women. The metabolic network verified significantly altered reactions and associated metabolites. Another study mapped proteomic and transcriptomic data from Alzheimer’s patients to Recon 1 [97]. This study firstly provided a network wide view of metabolic alterations associated with AD progression, and also identified novel sets of drug targets and biomarkers for AD. In reality, one of the first applications of Recon 1 was to interpret the metabolic effects of gastric bypass surgery through mapping human skeletal muscle gene expression data onto the metabolic network [98]. Another study integrated Recon 1 and EHMN with skeletal muscle transcriptomic data to identify Type 2 diabetes metabolic signatures utilizing two expression datasets [85]. These encompassed reported metabolites and transcription factors with significant enrichment of binding sites in enzyme-coding genes.

### 3.2. Metabolic Networks in Disease Prediction and Diagnosis

Metabolic networks have great potential in disease prediction and diagnosis. Metabolic network analysis can identify changes in metabolite concentrations, metabolic pathways, or metabolic enzymes that are associated with specific diseases. Biomarkers refer to biochemical indicators, which can signify possible changes in the function or structure of cells, tissues, organs, and systems. They are discriminant features related to the onset and progression of disease [99]. Metabolites have long been used as biomarkers in blood or urine to diagnose disease. Metabolic biomarkers refer to metabolites or combinations of metabolites associated with a particular disease. By comparing the metabolic profiles of diseased and healthy groups, metabolite pairs that change during disease onset and progression can be identified. These can elucidate pathogenesis and serve as early diagnosis biomarkers or for evaluating treatment efficacy [100].

Chang et al. [99] constructed sex-specific and apolipoprotein E (APOE)-specific metabolic networks. They proposed patient-specific biomarkers predictive of disease state and significantly associated with cognitive function. Based on computational network modeling, they integrated cognitive assessments and metabolomic profiling to confirm targeted precision therapeutics for Alzheimer’s disease (AD) patient subgroups. Recently, a bi-random walks method predicted disease–metabolite associations by executing the algorithm on reconstructed networks [101]. 

Furthermore, metabolic network analysis can predict disease progression. By analyzing dynamic changes in metabolic network models, researchers can simulate disease progression and predict the progression rate and possible outcomes [102]. This elucidates disease occurrence mechanisms and provides important guidance for disease treatment and intervention. 

Metabolic network analysis plays an important role in cancer research. Tumors reprogram biochemical pathways to promote unregulated cell growth and survival [103]. Metabolic network facilitates the discovery of specific metabolic dependencies that arise in cancers [104]. The complex interrelationships between oncogenes, gene expression, and metabolism offer the potential to discover novel biomarkers and drug targets with therapeutic and prognostic value. Dai et al. [105] used a novel unbiased correlation-based network analysis to discover crucial genes and metabolites in breast cancer metabolism. In this study, they identified both gene and metabolic hubs with prognostic value in breast cancer patients by effectively integrating gene expression and metabolomics data. The identification of crucial novel regulators of breast cancer supports the use of correlated network analysis to analyze large metabolic datasets and uncover treatment opportunities.

On the other hand, tumor heterogeneity is a considerable limitation in cancer treatment. Metabolic networks can help stratify cancer patients, gain biological insights into subtypes, and ultimately identify subtype-specific therapeutic targets. Previous research mainly relied on key genes or metabolites and their expression levels. Another study on hepatocellular carcinoma focused on network heterogeneity itself rather than molecular features or known signatures to achieve tumor stratification [106]. In this study, hepatocellular carcinoma tumors were stratified into three distinct subtypes based on an entire metabolic network-driven method. These subtypes showed considerable differences in clinical survival associated with WNT/β-catenin-associated lipid metabolism, altered kynurenine metabolism, and PI3K/AKT/mTOR signaling.

In conclusion, metabolic network analysis has broad potential applications in disease prediction and diagnosis (Table 2). By revealing the interactions and regulatory mechanisms between metabolites, analyzing metabolic abnormalities and metabolic markers, and predicting disease progression, metabolic network analysis provides an important theoretical framework and research methodology for disease prediction and diagnosis.

### 3.3. Drug Discovery and Disease Treatment

**Figure 7:**
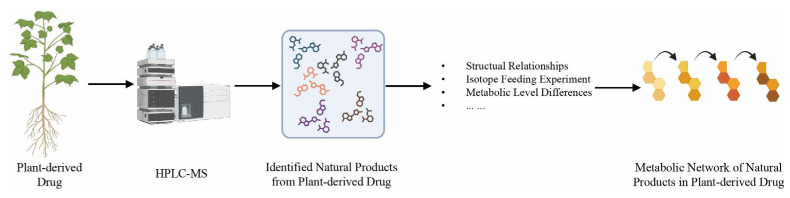
Metabolic networks provide insight into the biosynthesis pathways of natural products in plant-derived drugs. Metabolic network analysis plays an important role in drug discovery and drug development. By studying the metabolic network, researchers can propose new biosynthesis pathways of natural products.

In addition, metabolic network analysis has become an invaluable tool for drug discovery and development. Studying metabolic networks allows researchers to predict a drug’s mechanism of action and metabolic fate [111]. Advances in systems biology enable the prediction of functional effects of system perturbations using large-scale network models. The topological features of metabolic networks confer flexibility and robustness to complex biosystems. And in general, they may explain why many drug candidates are ineffective and why unexpected severe side effects happen [112]. Understanding these network properties is essential for rational drug design to improve efficacy and reduce adverse effects. Metabolic network models have been applied to simulate drug treatment and predict side effects. Chang et al. [113] reconstructed a kidney cell network to investigate toxicity of a withdrawn drug. By removing off-target metabolic enzymes from the model, they predicted the affected metabolic pathways and fluxes, elucidating the drug’s side effects. Metabolic networks have also provided insights into biosynthetic pathways for plant-derived natural product drugs (Figure 7). One study constructed an alkaloid network from Macleaya to propose new pathways for several compounds [114]. Therefore, metabolic network analysis is a useful tool for simulating drug effects, predicting adverse events, and elucidating biosynthetic pathways of natural product drugs.

Another advantage of metabolic network analysis is the ability to narrow down putative drug targets for in vitro validation, reducing reliance on expensive and time-consuming experimental approaches [115]. By analyzing crucial nodes and regulatory pathways in metabolic networks, key molecules in disease processes can be identified as potential therapeutic targets or lead compounds. These may include important metabolic regulators, bottleneck enzymes, and transporters, or disease-associated metabolites. Recent years, modeling cancer metabolism has been widely used in metabolic networks [96]. Tissue-specific and generic models have allowed prediction of drug targets in cancers [116,117]. Comparing healthy metabolic networks and cancer networks reveal cancer-specific features which could be potential pan-cancer targets [76]. Kanhaiya et al. [118] used an enzyme-centric metabolic network model of breast cancer to pinpoint drug targets. Moreover, metabolic network analysis has been applied to identify effective drug targets and develop novel antimicrobials. One antimicrobial drug development strategy involves determining inhibitors of key biological pathways in pathogens [119,120]. Bacterial metabolism presents many potential drug targets. Genome-scale reconstructions of bacterial metabolic networks have, therefore, become computational platforms for identifying target enzymes [121,122]. More recently, genome-scale metabolic network (GMN) models have enabled systems-wide investigations of pathogen and host metabolism [123,124]. One network-based metabolism-centered study simulated the *K. pneumoniae* MGH 78578 metabolism through the GMN to determine effective drug targets [125]. Potential inhibitors were further identified by virtual screening and synthetic lethality analysis. A similar framework integrating metabolic networks and virtual screening was applied to find effective drugs against *E. coli* [126]. In this study, they finally suggested eight FDA-approved antimicrobials as potential drugs to target the essential genes found. Therefore, metabolic network analysis is a powerful tool for elucidating pathogen metabolism, finding therapeutic targets, and developing novel antimicrobials.

Ultimately, metabolic network analysis represents a crucial tool for improving drug testing and optimizing therapeutic strategies [127]. By analyzing metabolic network models, researchers can evaluate the effects of drugs on specific metabolic pathways, predict drug efficacy and adverse reactions, and optimize drug dosing and administration regimens. This facilitates improved drug efficacy, reduced side effects, and enables individualized treatments tailored to each patient, thereby making drug treatments more accurate and beneficial to the patients. Exploiting synthetic lethality is a hopeful strategy for discovering targeted cancer therapies. Metabolic networks have proven highly effective at identifying synthetic lethal (SL) interactions. One study integrated networks and mutation data to detect synthetic lethal pairs across cancer types, providing insights for novel targeted therapies [128]. 

In general, identifying drug targets in biological networks requires controlling disease-associated nodes (e.g., genes, enzymes, metabolites) at minimal cost [129]. Compared to protein–protein interaction networks (PPN), metabolic networks have advantages for drug discovery as they can be used to uncover novel functional links between genes, revealing disease or drug-associated gene candidates. However, the exact functions of these novel genes require further experimental elucidation. In contrast, the functions of all proteins in metabolic networks are known [68]. The key goal is to systematically analyze metabolic networks to understand the whole system in relation to disease and identify potential targets.

## 4. Future Directions

The development of individualized metabolic networks represents an important future direction in the field. Currently, metabolic networks are generally constructed based on group-level metabolic covariance rather than individual-level connectivity, and therefore, cannot be directly used for individualized disease prediction [130]. However, metabolic differences between individuals can be significant. One potential solution is to integrate genetic, phenotypic, and environmental data to construct more accurate personalized metabolic networks. This approach would require more precise measurement techniques and robust individual-level data to delineate interindividual metabolic differences. As technology advances, the construction of individualized metabolic networks that incorporate genetic, phenotypic, and environmental factors may enable more accurate disease prediction and treatment at the individual level.

The development of robust metabolic networks will require expanded data sharing and collaboration. Currently, the available metabolomics data are limited, which restricts the construction of comprehensive networks. Additionally, issues with data quality, including measurement errors and sample contamination, can lead to incomplete or inaccurate networks that have limited repeatability and reliability in application. To enhance metabolic network construction, it will be critical to improve the accuracy of data acquisition and measurement techniques. Furthermore, strengthened data sharing and cooperation initiatives are needed to enrich metabolic network databases. By facilitating open access to high-quality metabolomics data, the research community can work collectively to develop more complete and reliable metabolic networks [131].

Moreover, optimization of the algorithms and models underlying metabolic network construction will be important to improve accuracy and interpretability. Traditional metabolic networks rely on correlation or pathway analyses, both of which have limitations. One future direction is to integrate correlation-based and pathway-based approaches to enable more complete elucidation of network structure and function. Additionally, metabolic network construction and analysis requires processing large, complex datasets. On the one hand, more powerful computational resources and efficient algorithms can help address the complexity of metabolic networks [132]. For example, parallel and distributed computing techniques could accelerate computations. On the other hand, novel algorithms and models that simplify network analysis and improve computational efficiency merit exploration. Recent artificial intelligence algorithms show promise for elucidating metabolic networks and enabling applications like predicting metabolic pathways, identifying drug-target interactions, and guiding individualized treatment [133,134,135]. Adopting these advanced computational approaches may expedite drug discovery and optimization by deriving more accurate insights from intricate metabolic network data.

Last but not least, investigating common network module dysregulation across different chronic diseases represents another important future research direction. It refers to the presence of common metabolic network module abnormalities in multiple chronic diseases. Current metabolic networks are often constructed to examine individual diseases. Identifying these shared network abnormalities could reveal common biological mechanisms and pathological processes. Comparing metabolic networks between diseases may highlight overlapping pathways and key regulatory nodes. Elucidating these disease connections and commonalities could provide new insights into disease etiology and progression. Moreover, targeting interventions to commonly dysregulated modules and regulatory points could enable more broadly applicable treatments. This approach may present new avenues for drug discovery and personalized medicine that can be leveraged across diverse conditions. Overall, delineating the shared network underpinnings among chronic diseases holds great promise. Research in this emerging area will likely provide comprehensive disease models, novel treatment strategies, and will propel the development of medical research and clinical practice.

## 5. Conclusions

In summary, further advancement in metabolic network analysis will require a multifaceted research effort. As technology continues to progress and in-depth studies elucidate the complexities of metabolic systems, metabolic network models can be expected to improve dramatically. Ongoing refinements in areas such as individualized network construction, the integration of diverse omics data, and the elucidation of shared network dysregulation among diseases will ultimately enhance the utility of metabolic networks across a wide range of biomedical applications. The future is promising for metabolic network analysis to fulfill its potential in accelerating disease prediction, diagnosis, prognosis, and precise treatment.

## Figures and Tables

**Table 1:** Codes for metabolic networks.

Metabolic Network	Method/Model	Language	Source
Correlation-based	Pearson correlation And Spearman rank correlation	Python	https://github.com/aishapectyo/Correlations-Pearson-Spearman (accessed on 28 November 2023)
	Distance correlation [20]	Python	https://github.com/vnmabus/dcor (accessed on 28 November 2023)
	Gaussian graphical model	R	https://github.com/donaldRwilliams/BGGM (accessed on 28 November 2023)
Causal-based	Causal inference model [21]	Python	https://github.com/BiomedSciAI/causallib (accessed on 28 November 2023)
	Structural equation model	R	https://github.com/yrosseel/lavaan (accessed on 28 November 2023)
	Dynamic causal model	Python	https://github.com/tmdemelo/pydcm (accessed on 28 November 2023)
Pathway-based	Pathway	Python	https://github.com/iseekwonderful/PyPathway (accessed on 28 November 2023)
Chemical structure similarity-based	Chemical structure similarity	Python	https://github.com/labsyspharm/lsp-cheminformatics (accessed on 28 November 2023)

**Table 2:** Metabolic network-based tools for disease study.

No.	Tool	Application	Character	URL
1	MAPPS [78]	A web-based tool for pathway prediction and network comparison, identification of potential drug targets	Allow users to upload custom data.	https://mapps.lums.edu.pk (accessed on 30 November 2023)
2	MetaboAnalyst [107]	A Network Explorer module for integrative analysis of metabolomics, metagenomics, and/or transcriptomics data.	For comprehensive metabolomic data analysis, interpretation, and integration with other omics data.	https://metaboanalyst.ca/ (accessed on 30 November 2023)
3	PathCase [108]	A database-enabled framework and Web-based computational tools for browsing, querying, analyzing, and visualizing stored metabolic networks.	Create a new metabolic network and/or update an existing metabolic network. The network can also be created from an existing genome-scale reconstructed network.	http://nashua.case.edu/PathwaysMAW/Web (accessed on 30 November 2023)
4	Met-express [109]	A powerful tool for uncovering novel therapeutic biomarkers.	Integrate a cancer gene co-expression network with the metabolic network to predict key enzyme-coding genes and metabolites in cancer cell metabolism.	None
5	Baumgartner C et al. [102]	A novel network-based approach for discovering dynamic metabolic biomarkers in cardiovascular disease.	Combine metabolic time-series data into a superimposed graph representation, highlighting the strength of the underlying kinetic interaction of preselected analytes.	None
6	Bidkhori et al. [110]	A metabolic network-based tool for identification and prioritization of anticancer targets.	Predict and rank potential anticancer non-toxic controlling metabolite and gene targets.	None

## Data Availability

Not applicable.
